# Supplementary material for: Dynamics of homeostats: the basis of electrical, chemical, hydraulic, pH and calcium signaling in plants
Source: Quant Plant Biol. 2025 Mar 21;6:e8. doi: 10.1017/qpb.2025.6 (PMC11950792; doi:10.1017/qpb.2025.6)
Supplement: Contador-Álvarez et al. supplementary material [file S2632882825000062sup001.docx]

**Supplementary Figure S1**


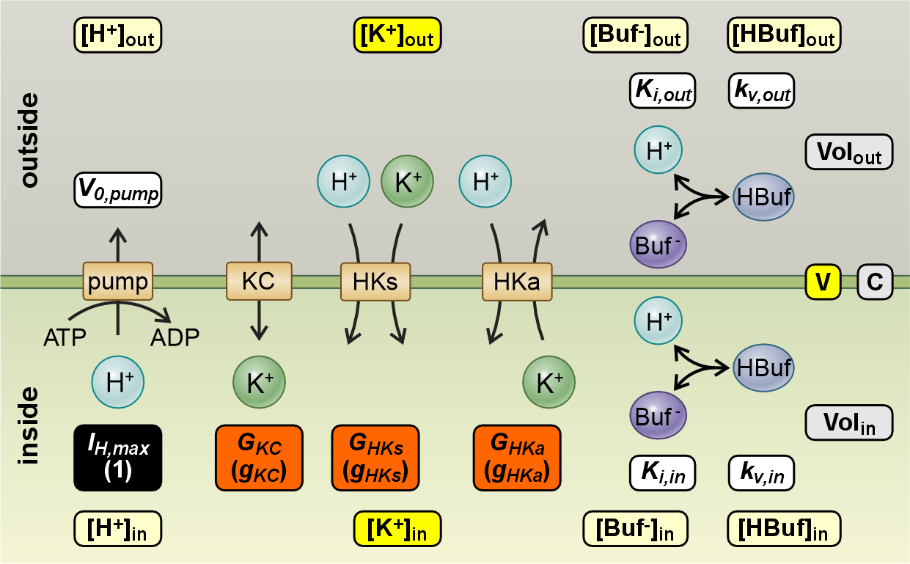


**Figure S1**. Overview of the K homeostat model. The K homeostat is a network of transporters that transport potassium in combination with the energizing proton ATPase. It consists of K^+^ channels (KC), H^+^/K^+^ symporters (HKs) and H^+^/K^+^ antiporters (HKa). All transporters are embedded in a membrane that separates the inside from the outside. The system is determined by the following parameters (see also Table S1): (i, *grey*) volume of the inside compartment (*Vol_in_*), volume of the outside compartment (*Vol_out_*) and the membrane capacitance (*C*) are determined by the geometry of the system. (ii, *black*) The maximal pump current (*I_H,max_*) was used to re-normalize the activities of the other transport processes (*G_X_* or *I_X_*: before re-normalization; *g_X_*: after re-normalization). (iii, *white*) The parameters *V_0,pump_*, *K_i,out_*, *K_i,in_*, *k_v,out_*, *k_v,in_* describe properties of the H^+^-ATPase and the pH-buffer reactions. They were set in this study. (iv, *orange*) The parameters *g_KC_*, *g_HKs_*, and *g_HKa_* describe the relative activities of the transporters (*g_X_* = *G_X_* / *I_H,max_*). (v, *yellow*) The remaining parameters [K^+^]_out_, [K^+^]_in_, [H^+^]_out_, [H^+^]_in_, [Buf^-^]_out_, [Buf^-^]_in_, [HBuf]_out_, [HBuf]_in_, and *V* depended on the setting of the homeostat and the pH-buffer reactions, which adjusted the respective ratios [K^+^]_out_/[K^+^]_in_, and [H^+^]_out_/[H^+^]_in_.

**Supplementary Figure S2**


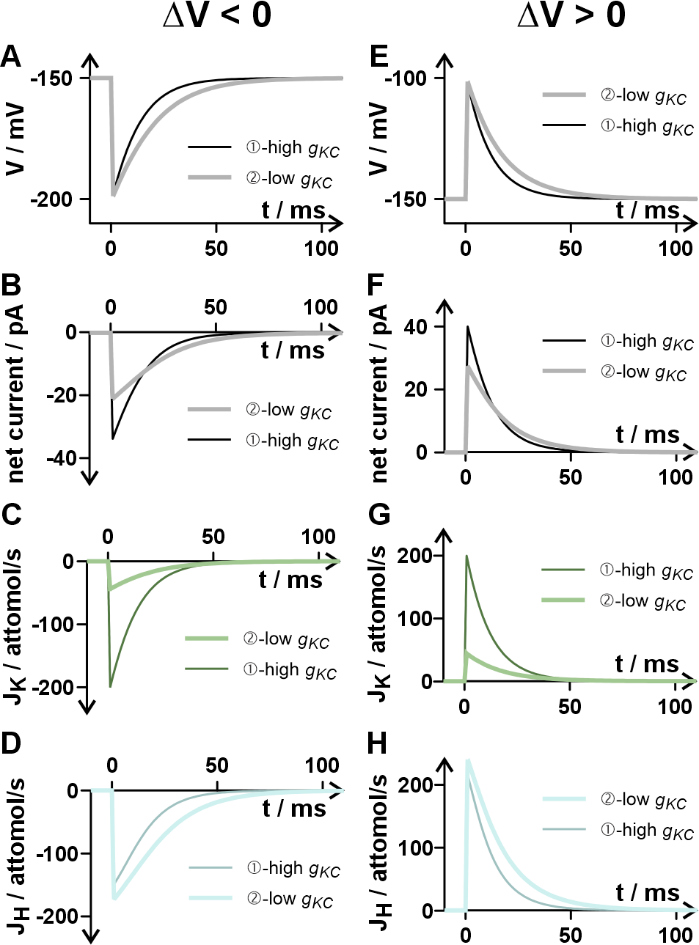


**Figure S2. Comparison of the reaction of K homeostats with different K-channel activities to a very rapid voltage pulse (ΔV)**. The parameter sets ➀: (*g_KC_* = 8 V^-1^, *g_HKs_* = 0.02 V-1, *g_HKa_* = 1.378 V^-1^) and ➁: (*g_KC_* = 0.8 V^-1^, *g_HKs_* = 0.474 V^-1^, *g_HKa_* = 0.77 V^-1^) represent two K homeostats with the same steady state values of $V_{ss}=-150mV$ and $E_{K}^{ss}=-115 mV$. The homeostat of scenario 2 has a ten-fold lower K-channel activity than that of scenario 1. The two K homeostats were kept in steady state and challenged at *t* = 0 s by a rapid (1 ms) hyperpolarization (A-D) or depolarization (E-H) and were then left to their own devices again. The induced net currents (B, F) cause the membrane voltage to reset over time (A, E). The net currents are carried by net fluxes of K^+^ (*J_K_*; C, G) and H^+^ (*J_H_*; D, H) across the membrane. Darker traces represent the K homeostat of scenario 1 with higher K-channel activity (➀-high *g_KC_*), while the light traces represent the K homeostat of scenario 2 with smaller K-channel activity (➁-low *g_KC_*).

**Supplementary Figure S3**


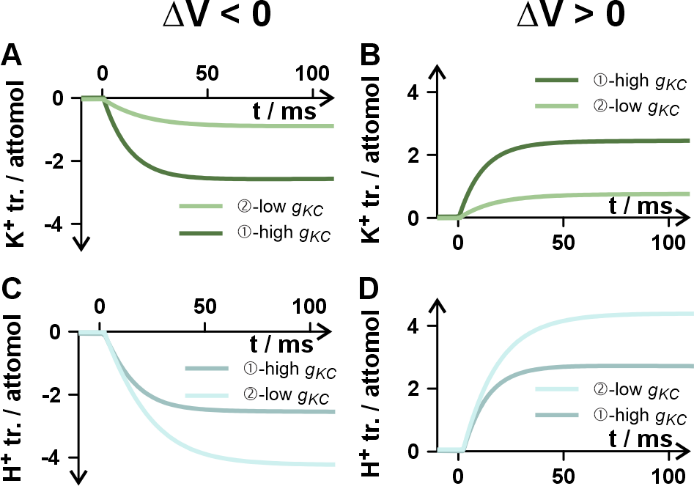


**Figure S3. Accumulated transmembrane net K^+^ and H^+^ transport during the relaxation process after a very rapid voltage pulse (ΔV)**. There are net K^+^ and H^+^ fluxes during the readjustment of the membrane voltage following a voltage stimulus (A, C: hyperpolarization, B, D: depolarization). Over time these fluxes sum up to a net transfer of K^+^ (A, B) and H^+^ (C, D) from the inside to the outside (positive values) or from the outside to the inside (negative values). Two K homeostats with the same steady state values of $V_{ss}=-150mV$ and $E_{K}^{ss}=-115 mV$ are represented by parameter sets ➀: (*g_KC_* = 8 V^-1^, *g_HKs_* = 0.02 V-1, *g_HKa_* = 1.378 V^-1^) and ➁: (*g_KC_* = 0.8 V^-1^, *g_HKs_* = 0.474 V^-1^, *g_HKa_* = 0.77 V^-1^). The homeostat of scenario 2 has a ten-fold lower K-channel activity than that of scenario 1. Darker traces represent the K homeostat of scenario 1 with higher K-channel activity (➀-high *g_KC_*), while the light traces represent the K homeostat of scenario 2 with smaller K-channel activity (➁-low *g_KC_*). The two K homeostats were kept in steady state and challenged at *t* = 0 s by a rapid (1 ms) hyperpolarization (-50 mV, A, C) or depolarization (+50 mV, B, D) and were then left to their own devices again.

**Supplementary Figure S4**


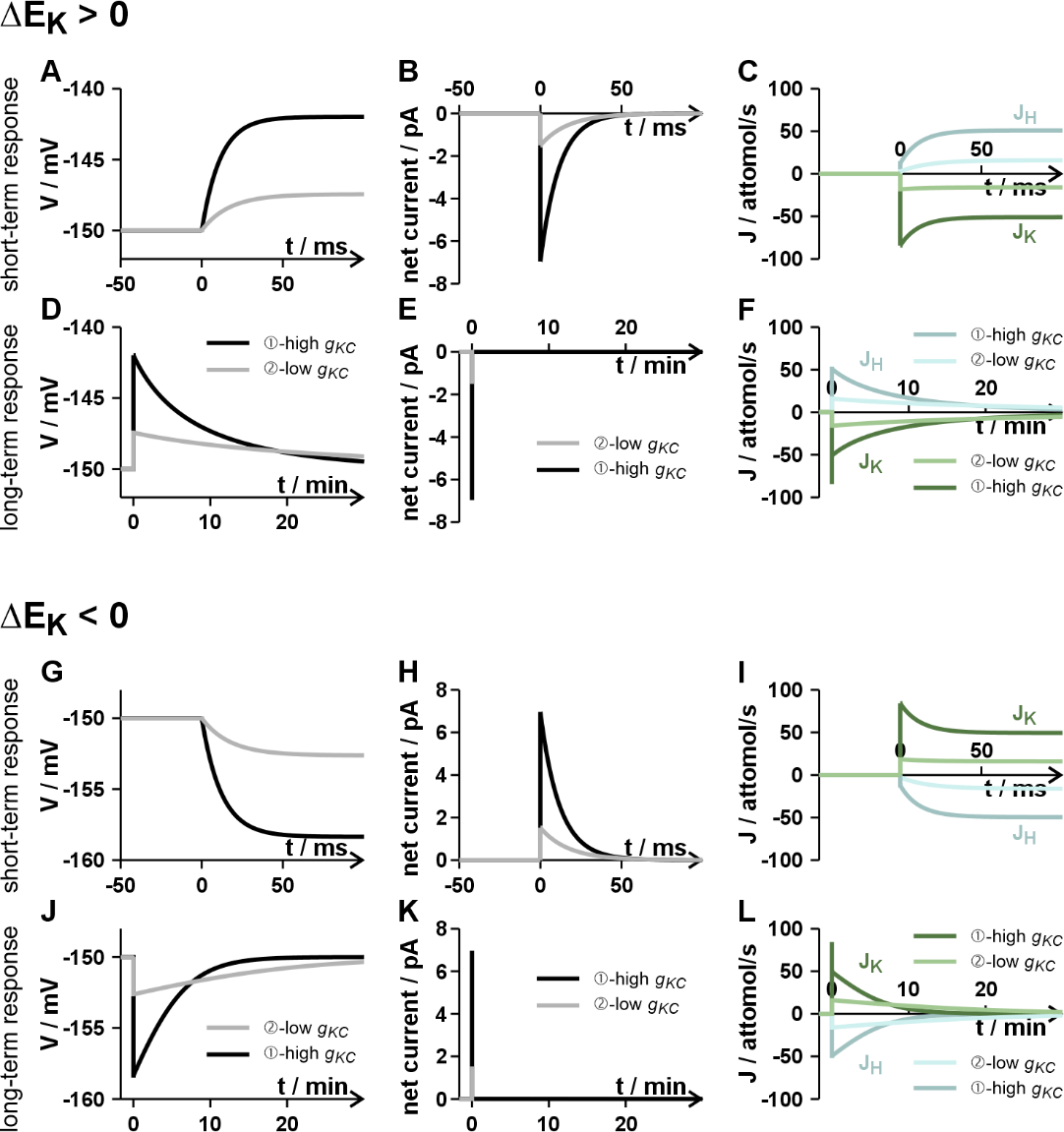


**Figure S4.** Response of the K homeostat to a rapid increase (A-F) or decreased (G-L) in the external K^+^ concentration. The K homeostat in steady state was brought out of equilibrium at *t* = 0 s by a doubling of [K^+^]_out_ (Δ*E_K_* > 0, A-F) or a halving of it (Δ*E_K_* < 0, G-L). The induced net currents (B, E, H, K) caused the membrane voltage to reset over time (A, D, G, J). The net currents were carried by net fluxes of K^+^ (*J_K_*, green) and H^+^ (*J_H_*, blue; C, F, I, L) across the membrane. Please note the different answers in the millisecond (short-term response, A-C, G-I) and minutes time scale (long-term response, D-F, J-L). The darker lines show the answer of the scenario ➀ homeostat with a higher K channel activity, while the brighter lines represent the answer of the scenario ➁ homeostat with a lower K channel activity.

**Supplementary Figure S5**


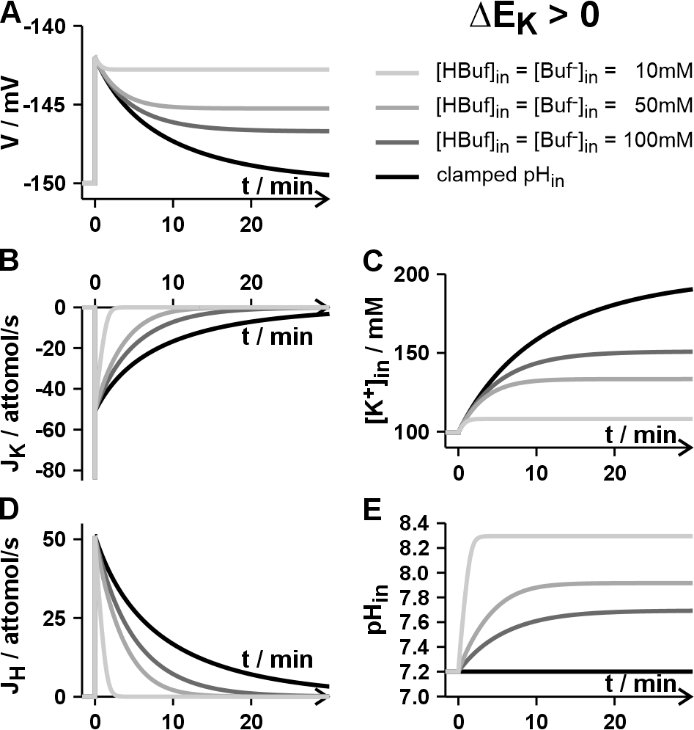


**Figure S5. Dependence of the long-term response of the K homeostat to a change in Δ*E_K_* on the internal pH-buffer capacity.** For illustration the K homeostat of scenario ➀ was brought out of equilibrium at *t* = 0 s by a doubling of [K^+^]_out_. Depending on the pH-buffer capacity at the inside, the adjustment of (A) the voltage *V*, (B) the potassium flux *J_K_*, and (D) the proton flux *J_H_* followed different time courses. The black lines indicate the situation when the pH was clamped. The lighter the lines are, the smaller the pH-buffer capacity was, represented by the concentrations [HBuf]_in_ and [Buf^-^]_in_. The smaller the buffer capacity, the faster pH_in_ changed (E) and the less [K^+^]_in_ accumulated (C).

Please note that in case of the buffer reaction $\left[ H^{+} \right]_{in}+\left[ {Buf}^{-} \right]_{in}\leftrightarrow\left[ HBuf \right]_{in}$ the pH_in_ value changes according to the “use” of the buffer. When 50% of the buffer capacity is exhausted, pH_in_ changes by ~0.5 pH units and when 80% of the buffer capacity is exhausted, pH_in_ changes by ~1 pH-unit. The difference of [K^+^]_in_ to 100 mM ([K^+^]_in_ – 100 mM) indicates the proton concentration that had to be replaced by the chemical buffer, *i.e.* the use of the buffer in mM.

**Table S1**

**Tab. S1.** Parameters of the homeostat system. The list of parameters determines the system of the K homeostat embedded in the membrane**.**

| Outside | - *Vol_out_* (pL): Volume of the outside compartment - [*H^+^*]*_out_* (µM): proton concentration at the outside - [*K^+^*]*_out_* (µM): K^+^ concentration at the outside - [Buf*^-^*]*_out_* (µM): concentration of the anionic base Buf^−^ of the pH-buffer at the outside - [HBuf]*_out_* (µM): concentration of the conjugate acid HBuf of the pH-buffer at the outside - *K_S,out_* (µM): dissociation constant of the buffer reaction at the outside - *k_v,out_* (µM^-2^⋅s^-1^): velocity constant of the buffer reaction at the outside |
| --- | --- |
| Membrane | - C (pF): Membrane capacitance - *V* (mV): membrane voltage - *I_H,max_* (pA): maximal cellular proton pump current re-normalized to $1={I_{H,max}}/{I_{H,max}}$ - *V_0,pump_* (mV): voltage, at which the pump current is zero fixed to *V_0,pump_* = -200 mV - *G_KC_* (pS): total conductivity of K^+^ channels re-normalized to $g_{KC}={G_{KC}}/{I_{H,max}}$ (V^-1^) - *G_HKs_* (pS): total conductivity of H^+^/K^+^ symporters  re-normalized to $g_{HKs}={G_{HKs}}/{I_{H,max}}$ (V^-1^) - *G_HKa_* (pS): total conductivity of H^+^/K^+^ antiporters re-normalized to $g_{HKa}={G_{HKa}}/{I_{H,max}}$ (V^-1^) |
| Inside | - *Vol_in_* (pL): Volume of the inside compartment - [*H^+^*]*_in_* (µM): proton concentration at the inside - [*K^+^*]*_in_* (µM): K^+^ concentration at the inside - [Buf*^-^*]*_in_* (µM): concentration of the anionic base Buf^−^ of the pH-buffer at the inside - [HBuf]*_in_* (µM): concentration of the conjugate acid HBuf of the pH-buffer at the inside - *K_S,in_* (µM): dissociation constant of the buffer reaction at the inside - *k_v,in_* (µM^-2^⋅s^-1^): velocity constant of the buffer reaction at the inside |
| Derived parameters | - $E_{H}=\frac{RT}{F}\cdot\ln\left( \frac{\left[ H^{+} \right]_{out}}{\left[ H^{+} \right]_{in}} \right)$ (Nernst potential for H^+^; mV) - $E_{K}=\frac{RT}{F}\cdot\ln\left( \frac{\left[ K^{+} \right]_{out}}{\left[ K^{+} \right]_{in}} \right)$ (Nernst potential for K^+^; mV) |

**Supplementary Text S1**

Change in driving force and fluxes due to perturbations

Changes in the membrane voltage ($\Delta V=V-V_{ss}$), the potassium gradient ($\Delta E_{K}=E_{K}-E_{K}^{ss}$), and/or the proton gradient ($\Delta E_{H}=E_{H}-E_{H}^{ss}$) affect the driving forces ($\Delta\mu$) and therefore the fluxes ($J$) across the transporters of the homeostat. To describe these effects mathematically, we expressed $J\left( \Delta\mu\right)$ by its Taylor series evaluated at ${\Delta\mu}_{ss}$, the electrochemical gradient in steady state conditions:

$J\left( \Delta\mu\right)=J^{ss}+\sum_{n=1}^{\infty} \left( \Delta\mu-{\Delta\mu}^{ss} \right)^{n}\cdot\frac{1}{n!}\cdot J^{\left( n \right)}$, (S1)

with $J^{\left( n \right)}=\frac{d^{n}}{\left( d\Delta\mu\right)^{n}}\left. J\left( \Delta\mu\right) \right|_{\Delta\mu={\Delta\mu}^{ss}}$ being the constant n^th^-derivative of $J\left( \Delta\mu\right)$ at $\Delta\mu={\Delta\mu}^{ss}$. Because the factor *n!* increases rapidly with *n*, the contribution of the various terms quickly decreases as *n* increases. To obtain very good insights for physiologically relevant gradients at most the second-order Taylor approximation (*i.e.*, not considering terms for *n* > 2) is reasonable (Dreyer, 2017):

$J\left( \Delta\mu\right)\approx J^{ss}+J^{\left( 1 \right)}\cdot\left( \Delta\mu-{\Delta\mu}^{ss} \right)\cdot\left[ 1+\frac{J^{\left( 2 \right)}}{2\cdot J^{\left( 1 \right)}}\cdot\left( \Delta\mu-{\Delta\mu}^{ss} \right) \right]$. (S2)

But usually, it is sufficient to consider the first-order approximation (**Figure S6**):

$J\left( \Delta\mu\right)\approx J^{ss}+J^{\left( 1 \right)}\cdot\left( \Delta\mu-{\Delta\mu}^{ss} \right)$. (S3)

In a further step, it can then be checked whether the error term

$Er=1+\frac{J^{\left( 2 \right)}}{2\cdot J^{\left( 1 \right)}}\cdot\left( \Delta\mu-{\Delta\mu}^{ss} \right)$ (S4)

has a serious impact on the result achieved. It should be noted that, with unchanged transporter activity, $J\left( \Delta\mu\right)$ is a monotonously increasing function, i.e. with increasing driving force $\Delta\mu$, $J$ increases but never decreases. Consequently, $J^{\left( 1 \right)}>0$. Please note that any potential decrease in an experimentally obtained flux curve (negative slope) is caused by effects on the transporter activity, *g_X_*, but not by an effect of the driving force $\Delta\mu$. Changes in *g_X_* are treated separately in our analyses.


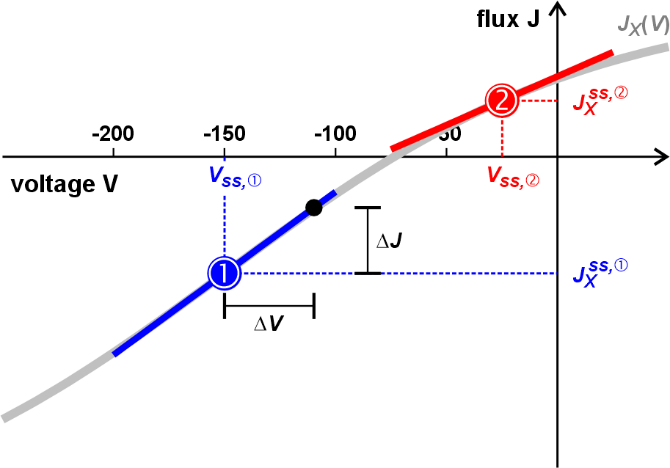
**Figure S6.** Linear approximation of the flux *J* to describe deviations from steady state. Two potential steady state conditions are shown (➀, blue, and ➁, red). Deviations from the respective steady state can be individually approximated by a linear curve (blue for deviations from ➀; red for deviations from ➁). For case ➀ it is illustrated how a change in voltage (Δ*V*) causes a change in flux (Δ*J*).

For a K^+^ selective channel the difference $\Delta\mu-{\Delta\mu}^{ss}$ is

${\Delta\mu}_{K}-{\Delta\mu}_{K}^{ss}=F\cdot\left( V-V_{ss}-\frac{RT}{F}\cdot\ln\left( \frac{\left[ K^{+} \right]_{out}}{\left[ K^{+} \right]_{in}} \right)+\frac{RT}{F}\cdot\ln\left( \frac{\left[ K^{+} \right]_{out}^{ss}}{\left[ K^{+} \right]_{in}^{ss}} \right) \right)=F\cdot\left( \Delta V-\Delta E_{K} \right)$. (S5)

Here, *R* is the gas constant, *F* the Faraday constant, and *T* the absolute temperature. In case of a 1:1 H^+^/K^+^ symporter, the difference is given by:

${\Delta\mu}_{HKs}-{\Delta\mu}_{HKs}^{ss}=F\cdot\left[ 2\cdot\left( V-V_{ss} \right)-\frac{RT}{F}\cdot\left\{ \ln\left( \frac{\left[ H^{+} \right]_{out}}{\left[ H^{+} \right]_{in}} \right)-\ln\left( \frac{\left[ H^{+} \right]_{out}^{ss}}{\left[ H^{+} \right]_{in}^{ss}} \right) \right\}-\frac{RT}{F}\cdot\left\{ \ln\left( \frac{\left[ K^{+} \right]_{out}}{\left[ K^{+} \right]_{in}} \right)-\ln\left( \frac{\left[ K^{+} \right]_{out}^{ss}}{\left[ K^{+} \right]_{in}^{ss}} \right) \right\} \right]$

$=F\cdot\left[ 2\cdot\Delta V-\Delta E_{H}-\Delta E_{K} \right]$. (S6)

And for a 1:1 H^+^/K^+^ antiporter it is:

${\Delta\mu}_{HKa}-{\Delta\mu}_{HKa}^{ss}=F\cdot\left[ \Delta E_{K}-\Delta E_{H} \right]$. (S7)

In the first order approximation, equation (S3) results for the K^+^ flux through a potassium channel:

$J_{K,KC}=J_{K,KC}^{ss}+s_{KC}\cdot\left( \Delta V-\Delta E_{K} \right)$, (S8)

with the positive constant $s_{KC}=F\cdot\frac{d}{d{\Delta\mu}_{K}}\left. J_{KC} \right|_{{\Delta\mu}_{K}={\Delta\mu}_{K}^{ss}}$. (S9)

The H^+^ and K^+^ fluxes through a 1:1 symporter are given by:

$J_{H,HKs}=J_{H,HKs}^{ss}+s_{HKs}\cdot\left( 2\cdot\Delta V-\Delta E_{H}-\Delta E_{K} \right)$, (S10)

$J_{K,HKs}=J_{K,HKs}^{ss}+s_{HKs}\cdot\left( 2\cdot\Delta V-\Delta E_{H}-\Delta E_{K} \right)$, (S11)

with the positive constant $s_{HKs}=F\cdot\frac{d}{d{\Delta\mu}_{HKs}}\left. J_{HKs} \right|_{{\Delta\mu}_{HKs}={\Delta\mu}_{HKs}^{ss}}$, (S12)

while the H^+^ and K^+^ fluxes through a 1:1 antiporter are given by:

$J_{H,HKa}=J_{H,HKa}^{ss}+s_{HKa}\cdot\left( \Delta E_{K}-\Delta E_{H} \right)$, (S13)

$J_{K,HKa}=-J_{H,HKs}=J_{K,HKa}^{ss}-s_{HKa}\cdot\left( \Delta E_{K}-\Delta E_{H} \right)$, (S14)

with the constant $s_{HKa}=F\cdot\frac{d}{d{\Delta\mu}_{HKa}}\left. J_{HKa} \right|_{{\Delta\mu}_{HKa}={\Delta\mu}_{HKa}^{ss}}$. (S15)

**
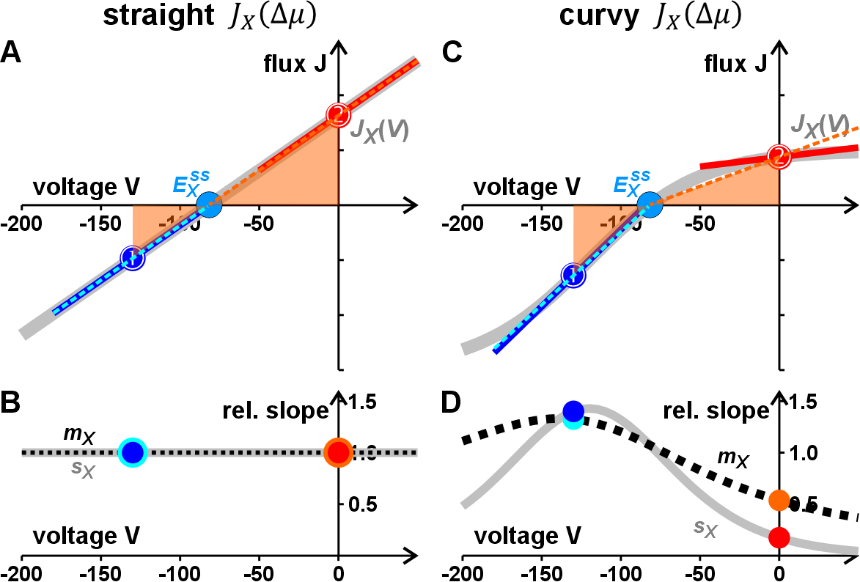
**

**Figure S7.** Comparison of linear approximations of $J_{X}\left( \Delta\mu\right)$ curves. Straight (A, B) and curvy (C, D) flux curves (grey curves in A and C) were represented in steady state by one point. For each curve, two potential steady state conditions are shown (➀, blue, and ➁, red). Deviations from any of these conditions could be linearly approximated as explained in Figure S6 (slope $s_{X}$, blue and red lines), but also by the slope $m_{X}$ of the orange triangles that were used to describe the $J_{X}^{ss}$ ordinate (dashed cyan lines for point ➀, and dashed orange lines for point ➁; see Figure 2 in Dreyer et al., 2024). (B, D) Comparison of $s_{X}$ (solid grey line, ➀, blue, and ➁, red) and $m_{X}$ (dashed black line, ➀, cyan, and ➁, orange) shows that they are identical in ranges where the $J_{X}\left( \Delta\mu\right)$ curve is straight. In curved sections, the difference between $m_{X}$ and $s_{X}$ is more pronounced with mostly $m_{X}>s_{X}$.

For a perfect linear relationship between $J$ and $\Delta\mu$ (**Figure S7A**), the $s_{X}$ values are identical to the $m_{X}=\frac{I_{Hmax}}{e_{0}}\cdot g_{X}$ values (**Figure S7B**). But also for curved $J_{X}\left( \Delta\mu\right)$ curves, the difference between $m_{X}$ and $s_{X}$ is moderate (**Figure S7D**). Curvatures in experimental $J_{X}\left( \Delta\mu\right)$ curves may originate from saturation effects of the transporter at very high driving forces. However, they can also be relicts of effects on the transporter activity $g_{X}$, which are difficult to separate in experiments. In order to make our theoretical approach practicable for illustrating the dynamic properties of homeostats in concrete examples, we considered linear $J_{X}\left( \Delta\mu\right)$ relationships and approximated $s_{X}\approx m_{X}>0$ in our numeric simulations. For further theoretical analyses, however, we continued to use the parameter $s_{X}$ to show that the conclusions from our simulations are solidly based on proper mathematical grounds.

For the numeric simulations, the following equations were applied. If changes in the driving force do not affect the activity of the K^+^ channel, *i.e.* parameter *g_KC_* remains unchanged, the new K^+^ flux through the potassium channels is given by [equation (12)]:

$J_{K,KC}=\frac{I_{Hmax}}{e_{0}}\cdot g_{KC}\cdot\left[ V_{ss}+\Delta V-\left( E_{K}^{ss}+\Delta E_{K} \right) \right]=J_{K,KC}^{ss}+\frac{I_{Hmax}}{e_{0}}\cdot g_{KC}\cdot\left( \Delta V-\Delta E_{K} \right)$. (S16)

If $\Delta V$, $\Delta E_{K}$, and $\Delta E_{H}$ do not affect the activity of the H^+^/K^+^ symporter, *i.e.* parameter *g_HKs_* remains unchanged, the altered H^+^ and K^+^ fluxes through a 1:1 symporter are given by [equations (13), (14)]:

$J_{K,HKs}=J_{K,HKs}^{ss}+\frac{I_{Hmax}}{e_{0}}\cdot g_{HKs}\cdot\left( 2\cdot\Delta V-\Delta E_{H}-\Delta E_{K} \right)$, (S17)

$J_{H,HKs}=J_{H,HKs}^{ss}+\frac{I_{Hmax}}{e_{0}}\cdot g_{HKs}\cdot\left( 2\cdot\Delta V-\Delta E_{H}-\Delta E_{K} \right)$. (S18)

If $\Delta V$, $\Delta E_{K}$, and $\Delta E_{H}$ do not affect the activity of the H^+^/K^+^ antiporter, *i.e.* parameter *g_HKa_* remains unchanged, the altered H^+^ and K^+^ fluxes through a 1:1 antiporter are given by [equations (15), (16)]:

$J_{K,HKa}=-\frac{I_{Hmax}}{e_{0}}\cdot g_{HKa}\cdot\left[ E_{K}^{ss}+\Delta E_{K}-\left( E_{H}^{ss}+\Delta E_{H} \right) \right]=J_{K,HKa}^{ss}-\frac{I_{Hmax}}{e_{0}}\cdot g_{HKa}\cdot\left( \Delta E_{K}-\Delta E_{H} \right)$, (S19)

$J_{H,HKa}=\frac{I_{Hmax}}{e_{0}}\cdot g_{HKa}\cdot\left[ E_{K}^{ss}+\Delta E_{K}-\left( E_{H}^{ss}+\Delta E_{H} \right) \right]=J_{H,HKa}^{ss}+\frac{I_{Hmax}}{e_{0}}\cdot g_{HKa}\cdot\left( \Delta E_{K}-\Delta E_{H} \right)$. (S20)

**Supplementary Text S2**

After disturbance of the steady state by a change in the membrane voltage (Δ*V*), in the K^+^ equilibrium (Δ*E_K_*) and/or in the H^+^ equilibrium (Δ*E_H_*), the following net fluxes are induced

Proton flux

$J_{H}=J_{HATPase}+J_{H,HKs}+J_{H,HKa}$ $=J_{HATPase}^{ss}+s_{ATPase}\cdot\Delta V+J_{H,HKs}^{ss}+s_{HKs}\cdot\left( 2\cdot\Delta V-\Delta E_{H}-\Delta E_{K} \right)+J_{H,HKa}^{ss}+s_{HKa}\cdot\left( \Delta E_{K}-\Delta E_{H} \right)$
$=\underset{=0, \text{eqn. }(11)}{\underbrace{J_{HATPase}^{ss}+J_{H,HKs}^{ss}+J_{H,HKa}^{ss}}}+\left( s_{ATPase}+2\cdot s_{HKs} \right)\cdot\Delta V+\left( s_{HKa}-s_{HKs} \right)\cdot\Delta E_{K}-\left( s_{HKs}+s_{HKa} \right)\cdot\Delta E_{H}$
$=\left( s_{ATPase}+2\cdot s_{HKs} \right)\cdot\Delta V-\left( s_{HKs}-s_{HKa} \right)\cdot\Delta E_{K}-\left( s_{HKs}+s_{HKa} \right)\cdot\Delta E_{H}$ (S21)

Taking into account the fact that the parameters $s_{X}>0$ (**Supplementary Text S1**), this means that

- 1. a depolarization ($\Delta V>0$) and/or a decrease of the proton gradient ($\Delta E_{H}<0$) cause a proton efflux ($J_{H}>0$),
  2. a hyperpolarization ($\Delta V<0$) and/or an increase of the proton gradient ($\Delta E_{H}>0$) cause a proton influx ($J_{H}<0$).
  3. The dependency of the proton flux on changes in the K^+^ gradient depends on the settings of the H^+^/K^+^ sym- and antiporters.
     - 1. With dominating symporters ($s_{HKs}>s_{HKa}$) an increase of the K^+^ gradient
          ($\Delta E_{K}>0$) causes a proton influx ($J_{H}<0$), while a decrease ($\Delta E_{K}<0$) provokes an efflux ($J_{H}>0$).
       2. With dominating antiporters ($s_{HKs}<s_{HKa}$) it is inverse. An increase of the K^+^ gradient ($\Delta E_{K}>0$) causes a proton efflux ($J_{H}>0$), while a decrease ($\Delta E_{K}<0$) provokes an influx ($J_{H}<0$).

Potassium flux

$J_{K}=J_{K,KC}+J_{K,HKs}+J_{K,HKa}$ $=J_{K,KC}^{ss}+s_{KC}\cdot\left( \Delta V-\Delta E_{K} \right)+J_{K,HKs}^{ss}+s_{HKs}\cdot\left( 2\cdot\Delta V-\Delta E_{H}-\Delta E_{K} \right)+J_{K,HKa}^{ss}-s_{HKa}\cdot\left( \Delta E_{K}-\Delta E_{H} \right)$
$=\underset{=0, \text{eqn. }(10)}{\underbrace{J_{K,KC}^{ss}+J_{K,HKs}^{ss}+J_{K,HKa}^{ss}}}+\left( s_{KC}+2\cdot s_{HKs} \right)\cdot\Delta V-\left( s_{KC}+s_{HKs}+s_{HKa} \right)\cdot\Delta E_{K}+\left( s_{HKa}-s_{HKs} \right)\cdot\Delta E_{H}$
$=\left( s_{KC}+2\cdot s_{HKs} \right)\cdot\Delta V-\left( s_{KC}+s_{HKs}+s_{HKa} \right)\cdot\Delta E_{K}-\left( s_{HKs}-s_{HKa} \right)\cdot\Delta E_{H}$ (S22)

This means ($s_{X}>0$):

- 1. a depolarization ($\Delta V>0$) and/or a decrease of the K^+^ gradient ($\Delta E_{K}<0$) cause a potassium efflux ($J_{K}>0$),
  2. a hyperpolarization ($\Delta V<0$) and/or an increase of the K^+^ gradient ($\Delta E_{K}>0$) cause a potassium influx ($J_{K}<0$).
  3. The dependency of the K^+^ flux on changes in the proton gradient depends on the settings of the H^+^/K^+^ sym- and antiporters.
     - 1. With dominating symporters ($s_{HKs}>s_{HKa}$) an increase of the proton gradient ($\Delta E_{H}>0$) causes a K^+^ influx ($J_{K}<0$), while a decrease ($\Delta E_{H}<0$) provokes an efflux ($J_{K}>0$).
       2. With dominating antiporters ($s_{HKs}<s_{HKa}$) it is inverse. An increase of the proton gradient ($\Delta E_{H}>0$) causes a K^+^ efflux ($J_{K}>0$), while a decrease ($\Delta E_{H}<0$) provokes an influx ($J_{K}<0$).

Total (charge) flux, current

$J_{total}=J_{H}+J_{K}=\frac{I}{e_{0}}=J_{HATPase}+J_{K,KC}+J_{K,HKs}+J_{H,HKs}+J_{K,HKa}+J_{H,HKa}$ $=J_{HATPase}^{ss}+s_{ATPase}\cdot\Delta V+J_{K,KC}^{ss}+s_{KC}\cdot\left( \Delta V-\Delta E_{K} \right)+J_{H,HKs}^{ss}+s_{HKs}\cdot\left( 2\cdot\Delta V-\Delta E_{H}-\Delta E_{K} \right)+J_{K,HKs}^{ss}$ $+s_{HKs}\cdot\left( 2\cdot\Delta V-\Delta E_{H}-\Delta E_{K} \right)+J_{H,HKa}^{ss}+s_{HKa}\cdot\left( \Delta E_{K}-\Delta E_{H} \right)+J_{K,HKa}^{ss}-s_{HKa}\cdot\left( \Delta E_{K}-\Delta E_{H} \right)$
$=\underset{=0, \text{eqn. }\left( 11 \right)}{\underbrace{J_{HATPase}^{ss}+J_{H,HKs}^{ss}+J_{H,HKa}^{ss}}}+\underset{=0, \text{eqn. }\left( 10 \right)}{\underbrace{J_{K,KC}^{ss}+J_{K,HKs}^{ss}+J_{K,HKa}^{ss}}}$ $+\left( s_{ATPase}+s_{KC}+4\cdot s_{HKs} \right)\cdot\Delta V-\left( s_{KC}+2\cdot s_{HKs} \right)\cdot\Delta E_{K}-2\cdot s_{HKs}\cdot\Delta E_{H}$
$=\left( s_{ATPase}+s_{KC}+4\cdot s_{HKs} \right)\cdot\Delta V-\left( s_{KC}+2\cdot s_{HKs} \right)\cdot\Delta E_{K}-2\cdot s_{HKs}\cdot\Delta E_{H}$ (S23)

$I_{total}=e_{0}\cdot J_{total}=e_{0}\cdot\left[ \left( s_{ATPase}+s_{KC}+4\cdot s_{HKs} \right)\cdot\Delta V-\left( s_{KC}+2\cdot s_{HKs} \right)\cdot\Delta E_{K}-2\cdot s_{HKs}\cdot\Delta E_{H} \right]$ (S24)

This means ($s_{X}>0$):

- 1. a depolarization ($\Delta V>0$), a decrease of the K^+^ gradient ($\Delta E_{K}<0$) and/or a decrease of the proton gradient ($\Delta E_{H}<0$) cause a positive net current ($I_{total}>0$),
  2. a hyperpolarization ($\Delta V<0$), an increase of the K^+^ gradient ($\Delta E_{K}>0$) and/or an increase of the proton gradient ($\Delta E_{H}>0$) cause a negative net current ($I_{total}<0$).

**Supplementary Text S3**

Adjustment of *V* and [K^+^] on two different timescales

The adaptations of the system to a disturbance were determined by the eqns. (17-24), which are of the type (i) ${d\left[ X \right]}/{dt}={\pm\left( Vol\cdot N_{A} \right)}^{-1}\cdot J_{x}$ or (ii) ${dV}/{dt}=-\left( e_{0}/C \right)\cdot J$. Changes in transporter activity, the membrane voltage or concentration gradients altered the ion fluxes, which in turn caused changes in the concentrations and the membrane voltage and ultimately resulted in a new steady state condition. While the fluxes *J_X_* or *J* (=*J_K_*+*J_H_*) [unit s^-1^] were similar in magnitude in both types of equation, the pre-factors $\left( Vol\cdot N_{A} \right)^{-1}$ [unit nM] and $e_{0}/C$ [unit mV] were different and specified the speed of the changes. To compare these pre-factors, the various parameters needed to be specified. The membrane capacity *C* ($=A_{M}\cdot1{\mu F}/{{cm}^{2}}=A_{M}\cdot{10}^{-14}F/{{\mu m}^{2}})$ is proportional to the surface of the membrane, *A_M_*, which could be expressed as $A_{M}=r_{SV}\cdot Vol$, with the surface to volume ratio of the cell, *r_SV_*. For a spherical cell with diameter *d* this ratio is $r_{SV}^{sphere}=6/d$ ($A_{M}^{sphere}=\pi\cdot d^{2}$, ${Vol}^{sphere}=\pi/6\cdot d^{3})$, and for a cubic cell with edge length *d* this ratio is also $r_{SV}^{cube}=6/d$ ($A_{M}^{cube}=6\cdot d^{2}$, ${Vol}^{cube}=d^{3})$. For a general estimation of the magnitude of the pre-factors, we could therefore approximate $A_{M}\approx6/d\cdot Vol$, where *d* [unit µm] indicates the dimension of the cell. With 1 L = 10^15^ µm^3^, *N_A_* = 6.022×10^14^ nmol^-1^, and *e_0_* = 1.6×10^-16^ mC the pre-factors in equations (17-24) could be expressed as:

$\frac{e_{0}}{C}=\frac{1.6\times{10}^{-16}mC}{{10}^{-14}\frac{F}{{\mu m}^{2}}\cdot\frac{6}{d}\cdot Vol}=2.7\times{10}^{-3}\cdot\frac{d}{\mu m}mV\cdot\frac{{\mu m}^{3}}{Vol}$ (S25)

$\frac{1}{Vol\cdot N_{A}}=\frac{nmol}{Vol\cdot\frac{L}{{10}^{15}{\mu m}^{3}}\cdot6.022\times{10}^{14}}=1.7nM\cdot\frac{{\mu m}^{3}}{Vol}$ (S26)

By calculating the ratio of these pre-factors, it can be deduced that a flux of one ion species which changes the membrane voltage by 1 mV causes a concentration change of ~63 nM if the cell dimension is *d* = 10 µm, while in a larger cell with a dimension of *d* = 100 µm the concentration change would be ~6.3 nM.

Thus, in a typical plant cell an additional flux of one ion species that caused a change in the membrane voltage by 10 mV, changed the concentration of the respective ion in the hundreds of nM range. In the case of K^+^ with [K^+^] = 1-100mM, a change in membrane voltage occurred very quickly compared to significant changes in concentration. *V* and [K^+^] therefore adapted on two different timescales: *V* fast and [K^+^] slow. However, the case was different for H^+^, considering the much lower proton concentrations. A pH value of 7.0, for instance, corresponds to [H^+^] = 100 nM. Here, the concentration changes would occur just as quickly as the voltage changes if there were no buffer systems. Nevertheless, buffer reactions of the type

$\text{H}^{\text{+}}+\text{Buf}^{\text{-}}\begin{matrix} k_{v} \\ \rightleftarrows\\ k_{v}\cdot K_{S} \end{matrix}\text{HBuf}$ (S27)

with an anionic base Buf^−^ and its conjugate acid HBuf, modified the pH responses. The capacity ($\left[ \text{Buf}^{\text{-}} \right]$, $\left[ \text{HBuf} \right]$) and dynamics (*k_V_*, *K_S_*) of the pH buffers thus primarily determined the changes in [H^+^] when the steady state was disturbed. In effect, the buffer reactions caused that the changes in [H^+^] were also slower than the changes in *V*.

**Supplementary Text S4**

Change in transporter activity is mathematically equivalent to a change in Δ*V*, Δ*E_K_* and Δ*E_H_*

In addition to modifications in the driving force, changes in the activity of the transporters, *i.e.* the parameters *g_KC_*, *g_HKs_*, and *g_HKa_*, also bring the homeostat out of equilibrium. Interestingly, the mathematical description of this case results in equations similar to equations (S16-S20), as will be shown in the following. In this case the disturbance of the system changes the parameter $g_{X}$ from the original steady state to $\tilde{g}_{X}$ that determines the new steady state condition. For a K^+^ channel, the new flux that has changed due to the disturbance is in general form

$J_{K,KC}=\frac{I_{Hmax}}{e_{0}}\cdot\tilde{g}_{KC}\cdot\left( V-E_{K} \right)+\underset{=0}{\underbrace{\left[ \underset{=\tilde{J}_{K,KC}^{ss}}{\underbrace{\frac{I_{Hmax}}{e_{0}}\cdot\tilde{g}_{KC}\cdot\left( V_{ss}-E_{K}^{ss} \right)}}-\frac{I_{Hmax}}{e_{0}}\cdot\tilde{g}_{KC}\cdot\left( V_{ss}-E_{K}^{ss} \right) \right]}}$ (S28)

with the altered parameter $\tilde{g}_{KC}$, the actual voltage *V*, the actual K^+^ gradient *E_K_*, the new steady state voltage *V_ss_*, the new steady state K^+^ gradient $E_{K}^{ss}$, and the new steady state flux $\tilde{J}_{K,KC}^{ss}$. The adding of 0 (in the brackets) has the technical reason that equation (S28) can now be re-ordered:

$J_{K,KC}=\tilde{J}_{K,KC}^{ss}+\frac{I_{Hmax}}{e_{0}}\cdot\tilde{g}_{KC}\cdot\left( V-V_{ss}-\left( E_{K}-E_{K}^{ss} \right) \right)=\tilde{J}_{K,KC}^{ss}+\frac{I_{Hmax}}{e_{0}}\cdot\tilde{g}_{KC}\cdot\left( \Delta V-{\Delta E}_{K} \right)$ (S29)

Identical considerations lead to

$J_{K,HKs}=\tilde{J}_{K,HKs}^{ss}+\frac{I_{Hmax}}{e_{0}}\cdot\tilde{g}_{HKs}\cdot\left( 2\cdot\Delta V-{\Delta E}_{H}-{\Delta E}_{K} \right)$ (S30)

$J_{H,HKs}=\tilde{J}_{H,HKs}^{ss}+\frac{I_{Hmax}}{e_{0}}\cdot\tilde{g}_{HKs}\cdot\left( 2\cdot\Delta V-{\Delta E}_{H}-{\Delta E}_{K} \right)$ (S31)

for 1:1 H^+^/K^+^ symporters, and to

$J_{K,HKa}=\tilde{J}_{K,HKa}^{ss}-\frac{I_{Hmax}}{e_{0}}\cdot\tilde{g}_{HKa}\cdot\left( \Delta E_{K}-\Delta E_{H} \right)$ (S32)

$J_{H,HKa}=\tilde{J}_{H,HKa}^{ss}+\frac{I_{Hmax}}{e_{0}}\cdot\tilde{g}_{HKa}\cdot\left( \Delta E_{K}-\Delta E_{H} \right)$ (S33)

for 1:1 H^+^/K^+^ antiporters.

Thus, comparison of equations (S16-S20) with (S28-S33) reveals their identical structures with the parameters $g_{KC}$ ($\tilde{g}_{KC}$), $g_{HKs}$ ($\tilde{g}_{HKs}$), and $g_{HKa}$ ($\tilde{g}_{HKa}$). A change in the parameter set (*g_KC_*, *g_HKs_*, *g_HKa_*) that sets new steady state values $\left( V_{ss} | E_{K}^{ss} \right)$ can therefore be interpret as a deviation of the system with Δ*V* and Δ*E_K_* from its steady state. An analogy might illustrate this relationship: The system is like a ball in a 3-dimensional landscape. For a disturbance of the system, we can change the position of the ball and keep the landscape fixed or we keep the position of the ball and move the landscape instead.
